# Antidepressant activity of ω-3 polyunsaturated fatty acids in ovariectomized rats: role of neuroinflammation and microglial polarization

**DOI:** 10.1186/s12944-020-1185-2

**Published:** 2020-01-08

**Authors:** Bin Wu, Qingen Song, Yongkang Zhang, Changshui Wang, Mengqi Yang, Jun Zhang, Wenxiu Han, Pei Jiang

**Affiliations:** 1 Department of Gynecology, Taian City Central Hospital, Taian, China; 2 Jinxiang People’s Hospital, Jining Medical University, Jining, China; 3 Institute of Clinical Pharmacy & Pharmacology, Jining First People’s Hospital, Jining Medical University, Jining, 272000 China; 4 Department of Pharmacy, The First Affiliated Hospital of Zhengzhou University, Zhengzhou, China

**Keywords:** Depression, Ovariectomy, Polyunsaturated fatty acids, Microglial polarization

## Abstract

**Background:**

Menopause predisposes individuals to affective disorders, such as depression, which is tightly related to neuroinflammation. While the neuroinflammatory condition has been demonstrated in ovariectomized (OVX) rodents, there is limited evidence concerning microglial polarization, a key process in brain immune activation, in menopause-related brain.

**Methods:**

Therefore, the present study aims to evaluate the polarized microglia in long-term OVX rats and we further explored whether supplementation of ω-3 polyunsaturated fatty acids (PUFA), the pleiotropic bioactive nutrient, is effective in the neurobehavioral changes caused by OVX.

**Results:**

Our data showed that OVX-induced anxiety and depression-like behaviors in rats, accompanied with increased neural apoptosis and microglial activation in the hippocampus. Additionally, OVX enhanced proinflammatory cytokines expression and suppressed the expression of anti-inflammatory cytokine, IL-10. Correspondingly, OVX reinforced NFκB signaling and shifted the microglia from immunoregulatory M2 phenotype to proinflammatory M1 phenotype. Meanwhile, daily supplementation with PUFA suppressed microglial M1 polarization and potentiated M2 polarization in OVX rats. In parallel, PUFA also exerted antidepressant and neuroprotective activities, accompanied with neuroimmune-modulating actions.

**Conclusion:**

Collectively, the present study firstly demonstrated the disturbed microglial polarization in the OVX brain and provide novel evidence showing the association between the antidepressant actions of PUFA and the restraint neuroinflammatory progression.

## Background

Menopause is strictly associated with affective disorders, whereas anxiety and depression are frequently-occuring and debilitating psychiatric diseases in menopause [1]. The occurrence menopausal disorders in both brain and periphery is related to the loss of ovarian function and estrogen deficiency. In this scenario, ovariectomized (OVX) rodents become a widely used animal model of menopause, which is generally referred to as surgical menopause [2]. Long-term after OVX, the animals develop a reliable predisposition to anxiety and depression-like behaviors [3]. Although OVX-induced hormonal deficiency is likely to be the cause of behavioral changes, the mechanisms underlying the brain pathological changes remain equivocal.

Neuroinflammation is recognized as a major contributor to depression. It has been reported that patients with depression are prone to have higher status of proinflammatory cytokines, including interleukin (IL)-1β, IL-6 and tumor necrosis factor α (TNFα) in the periphery and central nervous system [4]. The stress-induced animal models of depression are also characterized with overproduction of proinflammatory mediators [5]. In support, treatment with the endotoxin, lipopolysaccharide (LPS), induces immune activation in both periphery and brain, resulting in depression-like behaviors [6]. Likewise, neuroinflammatory provocation has been repeatedly observed in OVX rodents, whereas antidepressant strategies, such as exercise, estrogen supplementation or inflammasome inhibition, also exerted immune-regulatory features [3, 7], strongly indicating a role for immune system in OVX-induced behavioral disturbance. Microglia is considered as the resident macrophage in the brain with a crucial role in neuroinflammatory progression. Like macrophage, microglia can polarize into proinflammatory M1 phenotype and immunoregulatory M2 phenotype, which is responsible for the production of proinflammatory or anti-inflammatory cytokines, respectively [8].

ω-3 polyunsaturated fatty acids (PUFA), the pleiotropic bioactive nutrient, contains anti-inflammatory and antidepressive activities [9]. Our previous researches demonstrated that PUFA can mitigate LPS-induced behavioral changes and restore overactivated neuroimmune function [10]. Although the neuroimmune-regulatory actions of PUFA has been found in various animal models, whether PUFA is effective in the immune activation induced by OVX remains unknown.

Therefore, the present study aims to evaluate phenotype of microlgia in the hippocampus of rats following long-term OVX and further to explore the immune-regulatory role of PUFA in the antidepressant mechanism.

## Materials and methods

### Animals

Female Sprague-Dawley rats about 12-week old were housed under a temperature- controlled (23 ± 2 °C) and 12/12 h light/dark cycle environment, with free access to food and water. All animal studies were carried out in accordance with the Regulations of Experimental Animal Administration issued by the State Committee of Science and Technology of the People’s Republic of China, with the approval of the Ethics Committee in Jining Medical University.

### PUFA supplementation and ovariectomy

The rats were randomly divided into four groups (*n* = 6–7): Sham-operated control group (Sham), PUFA, OVX and OVX + PUFA. Animals were bilaterally ovariectomized under anesthesia with sodium pentobarbital (50 mg/kg) through intraperitoneal injection. Following two small incisions, the ovaries, oviducts and top of the fallopian tubes were bilaterally clamped and removed in OVX group. After anesthesia, similar protocols were conducted in Sham group with the abdominal wall opened and the ovaries exteriorized but not removed to create similar stressful events. Refined fish oil was administrated daily by gavage (1.5 g/kg) in PUFA and OVX + PUFA groups for PUFA treatment (approximately 340 mg/g for EPA, 240 mg/g for DHA, Sheng Tianyu Biotechnology, China) at the same day before OVX surgery. The treatment procedures lasted for 10 weeks before sacrifice. The dosage of PUFA was selected based on our preliminary research and previous experiment showing the robust antidepressant effect of PUFA [10, 11]. The animals in Sham group were sacrificed at the diestrus phase to avoid the effects of estrus cycle. At the end of the 10 weeks, behavioral tests were performed and the rats were anesthetized with sodium pentobarbital (50 mg/kg). Blood was collected and the brain was quickly removed on the ice after cardiac perfusion with PBS. The left hemisphere of the brain was maintained in 4% paraformaldehyde and then embedded in paraffin, prepared for histopathological examination and immunofluorescent staining. For the right hippocampus, the tissue was dissected and homogenized, which was used for and Western blot and PCR analysis.

### Behavioral tests

Elevated plus maze (EPM) test, a validated method to assess axiety-like behavior, was used in the present study [12]. In brief, the maze apparatus was a cross-shaped Plexiglas platform with two opposite open arms (OA, 50 × 10 cm) and two opposite closed arms (CA, 50 × 10 cm), connected by a central platform (CP, 10 × 10 cm). The rats were placed at the center of the maze with the head facing one of the open arm. The observer should be at least 1 m away from the maze. Using the video camera mounted vertically above the maze to record the number of entries into the open and closed arms, and the time spent in each arm during the 5 min period.

The paradigm of forced swim test (FST) is based upon the evaluation of immobility, as a measure of behavioral despair in stressful and inescapable situations [13]. In brief, each rat was placed in a glass cylinder (45 cm height, 25 cm diameter) which contained approximately 35 cm of water (24 ± 1 °C) for 15 min. The rats were then dried and transfered to their cage. They were placed again in same cylinders 24 h later for a 5 min swim test while it was videotaped. And the duration of immobility which defined as floating passively and only making slight movements to keep the head above water, was scored by an experienced observer blind to the experiment design.

Sucrose Preference Test (SPT) is diffusely used for the measurement of the depressive-like behavior in rats [14]. Prior to SPT, all the rats were housed individually. After two bottles of 1% sucrose solution placed on each cage for 48 h, rats were deprived water for 14 h. Then two pre-weighing bottles, one containing 1% sucrose solution and the other containing water, were placed to each rat. The side of the two bottles was randomly placed to reduce spatial bias and the bottles were weighed again after 1 h. The weight difference was deemed to be the rat intake from each bottle. The preference for sucrose was measured as a percentage of the consumed 1% sucrose solution relative to the total amount of liquid intake.

The novelty-suppressed feeding test (NSFT) was adapted from previous study [15]. Before testing, rats were fasted for 24 h. The rats were placed in an open field (75 × 75 × 40 cm). And a white paper (10 × 10 cm) with a small amount of food was placed in the center of open field. Rats were allowed to move freely for 8 min in open field. Using a stopwatch, record the latency to feed, specifically the time it took for the rat to approach and take the first bite of the food. Immediately afterwards, the animals were transferred to their cage, and the total food intake belong the next 5 min was also weighed to avoid the influence of the appetite of rats.

### Hormone assay

Serum were immediately separated by centrifuging at 4000 rpm for 10 min at 4 °C and stored at − 80 °C until assay. The serum concentrations of 17β-estradiol were quantified using a commercial enzyme-linked immunosorbent assay (ELISA) kit (Guxi Biotech, China) according to the manufacturer’s protocol.

### Apoptosis analysis

The tissues were fixed in 10% phosphate-buffered paraformaldehyde for 48 h and then embedded in paraffin which prepared for immunohistochemical staining and histopathological examination. The presence of apoptosis was assessed by the terminal deoxynucleotidyl transferase-mediated FITC-dUTP nick end labeling (Tunel) method, which detects fragmentation of DNA in the nucleus during apoptotic cell death in situ. The apoptosis detection assay was performed using a commercially available kit following the manufacturer’s protocol (Keygen Biotech, Nanjing, China). Three sections per mouse were used and the images of the hippocampal dentate gyrus (DG) region were analyzed.

### Immunostaining

For the immunofluorescent analysis, paraffin-embedded cross sections of the tissues (6 μm thickness) were dewaxed in xylol, rehydrated, antigen retrieval by boiling citric acid buffer (0.01 mol/L, pH 6.0) and rinsed in PBS. Then tissue sections were incubated with blocking 5% goat serum at 25 °C for 1 h. For immunocytochemistry, cells grown on coverslips were fixed with ice-cold 4% paraformaldehyde in PBS for 10 min. Cells were blocked using 1% BSA + 0.3% (v/v) Triton X-100 + 0.3 M glycine (1% BSA) in PBS at 25 °C for 45 min. The sections were then incubated with the primary antibody, anti-ionized calcium-binding adapter molecular 1 (Iba-1) (Abcam, 1:200). The sections were washed with PBS three times and stained with DAPI (Beyotime Biotechnology, China) to stain the cell nuclei. Immunofluorescent images were taken with an inverted fluorescence microscope (Olympus, Japan) and analyzed by using the Image J software to obtain the mean fluorescence density of each visual field.

### Western blot analysis

For western blotting analysis, total protein was prepared and the concentrations were analyzed using Bradford method. Samples were loaded on precast 12% SDS-PAGE gels with 50 μg protein in each lane. Proteins in the gels were transferred to a PVDF membrane and blocked for 1 h in 5% non-fat dry milk in TBS-T (25 mM Tris, pH 7.5, 150 mM NaCl, 0.05% Tween-20). The following antibodies and concentrations were used over night at 4 °C; phosphorylation of NF-κB p65 subunit (p-p65, Proteintech, 1:500), p65 (Proteintech; 1:800), NF-κB inhibitor (IκB, Abcam, 1:1000), iNOS (Proteintech; 1:500), Arginase-1 (Arg-1, Proteintech, 1:30000) and β-actin (Proteintech; 1:4000). After that, it was probed with HRP-conjugated secondary antibody for 40 min. The signals were digitally scanned and was quantified using Image J software. The signals were normalized to β-actin as an internal standard.

### Real-time PCR analysis

Total RNA was extracted by using Trizol reagent (invitrogen, USA) following the manufacturer’s instructions. The RNA concentration and integrity was determined by spectrophotometry (260–280 nm) (Jingke, Ningbo, China). cDNA was generated from total RNA using the Revert Aid First Strand cDNA Synthesis Kit (Thermo Fisher Scientific, Tewksbury, MA, USA). Quantitative PCR was performed using gene-specific primers (Table 1) and SYBR green PCR kit (Applied Bio-systems, USA) on Bio-rad Cx96 Detection System (Bio-rad, USA). All PCR experiments was tested in triplicate. The PCR amplification program were: 50 °C for 2 min, 95 °C for 10 min, 40 cycles of amplification at 95 °C for 15 s and 60 °C for 1 min. Relative quantitation for PCR product was normalized to β-actin as an internal standard.

**Table 1:** Primer sequences used for the qPCR analysis

Gene	Sense Primer (5′-3′)	Antisense Primer (5′-3′)
IL-1β	AGGTCGTCATCATCCCACGAG	GCTGTGGCAGCTACCTATGTCTTG
IL-6	CACAAGTCCGGAGAGGAGAC	ACAGTGCATCATCGCTGTTC
TNFα	GAGAGATTGGCTGCTGGAAC	GAGAGATTGGCTGCTGGAAC
IL-10	GTTTTACCTGGTAGAAGTGATGCC	CCACTGCCTTGCTTTTATTCTC
IL-4	ACAGGAGAAGGGACGCCAT	GAAGCCCTACAGACGAGCTCA
CD86	TAGGGATAACCAGGCTCTAC	CGTGGGTGTCTTTTGCTGTA
iNOS	AGTGGCAACATCAGGTCGG	CGATGCACAACTGGGTGAAC
CD206	AGTTGGGTTCTCCTGTAGCCCAA	ACTACTACCTGAGCCCACACCTGCT
Arg-1	GGGAAAAGCCAATGAACAGC	CCAAATGACGCATAGGTCAGG
β-Actin	CATCCTGCGTCTGGACCTGG	TAATGTCACGCACGATTTCC

### Statistical analysis

All statistical procedures were performed on SPSS version 18. Data were expressed as mean ± SD. Differences between groups were determined by two-way ANOVA. The prior level of significance was established at *p* < 0.05. Tukey’s test for post hoc comparisons were performed following any significant ANOVA.

## Results

### Effects of PUFA on body weight, uterine weight, estradiol and behaviors

**Fig. 1:**
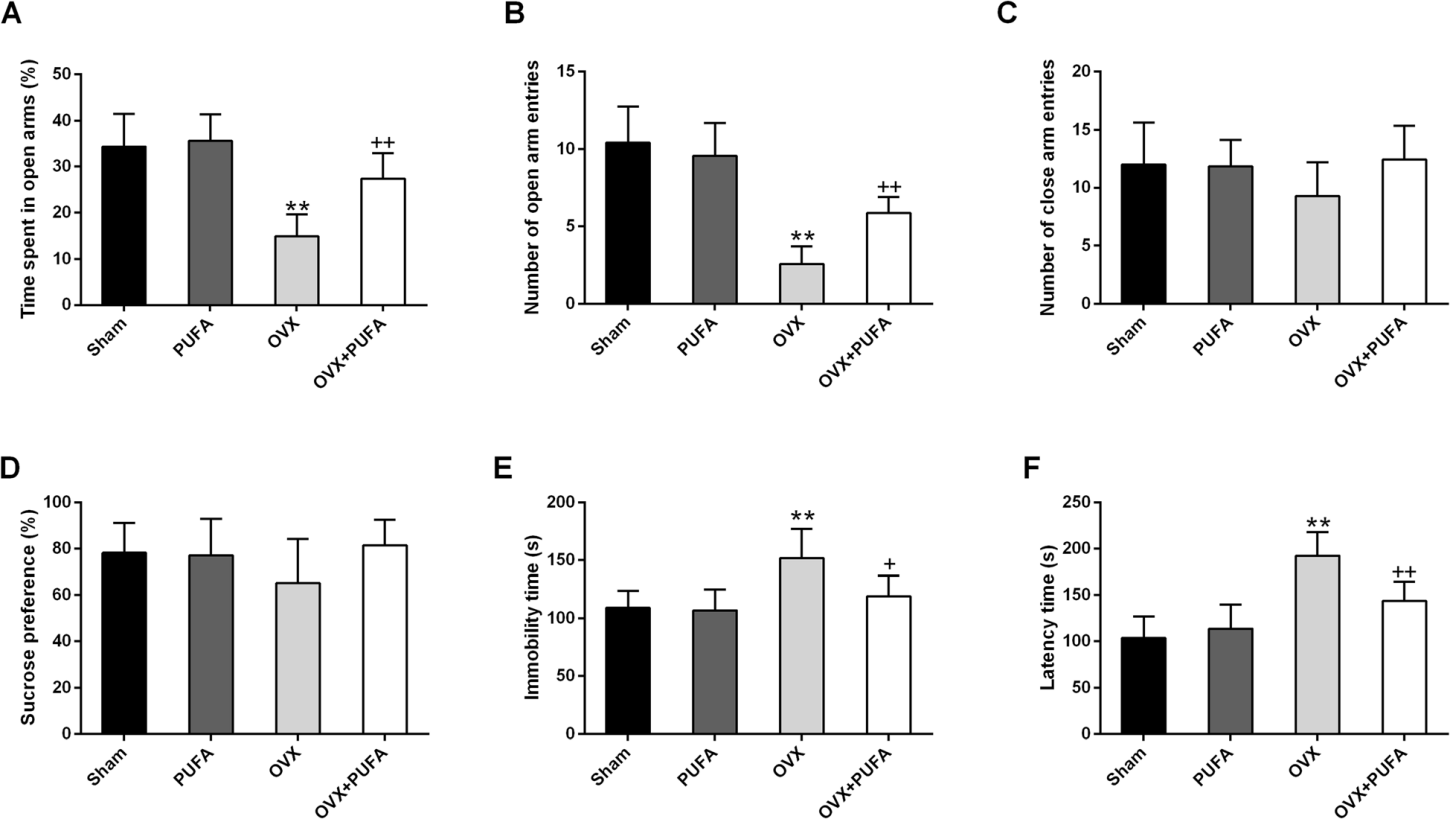
Alleviating effects of PUFA on OVX-induced behavioral changes. Anxiety-like behavior was assessed by elevated plus maze (EPM) test (**a**-**c**) and depression-like behavior was assessed by sucrose preference test (SPT) (**d**), forced swim test (FST) (**e**) and novelty-suppressed feeding test (NSFT) (**f**). Data are means ± SD (*n* = 6–7). ^**^ *p* < 0.01 compared to Sham group. ^+^ *p* < 0.05, ^++^ *p* < 0.01 compared to OVX group

As shown in Table 2, OVX markedly decreased circulating estradiol status (main effect of OVX, F = 82.77, *p* < 0.01) and uterine weight (main effect of OVX, F = 73.52, *p* < 0.01), indicating that the animal model was successfully built. In line with previous findings [16], OVX pronouncedly increased the body weight growth (main effect of OVX, F = 41.03, *p* < 0.01). However, PUFA treatment only slightly but non-significantly increased estradiol levels with no significant effect on body weight gain and uterine weight. Nonetheless, PUFA exerted robust anti-anxiety properties in EPM test of OVX rats and there was a significant interaction between PUFA and OVX on anxiety-like behaviors in time spent in open arms (F = 16.09, *p* < 0.01) (Fig. 1a) and open arm entries (F = 24.73, *p* < 0.01) (Fig. 1b). In addition, although both PUFA and OVX didn’t affect sucrose preference in SPT (Fig. 1d), PUFA alleviated OVX-induced depressive-like behaviors with decreased immobility time in FST (PUFA×OVX interactions, F = 5.82, *p* < 0.05) (Fig. 1e) and latency time to feed in NSFT (PUFA×OVX interactions, F = 12.41, *p* < 0.01) (Fig. 1f).

**Table 2:** Effect of ω-3 PUFAs supplementation on body weight changes, uterine weight and serum estradiol status in ovariectomized (OVX) rats

Group	Body weight gain (g)	Uterine weight (mg)	Estradiol (pg/ml)
Control	227.49 ± 12.22	621.68 ± 106.31	106.06 ± 9.97
PUFA	231.19 ± 19.61	592.19 ± 77.09	117.77 ± 12.03
OVX	278.95 ± 10.92**	155.84 ± 32.62**	29.26 ± 5.37**
OVX + PUFA	283.34 ± 12.66	161.25 ± 48.07	35.04 ± 7.52

Data are means ± SD (*n* = 7–9). ** *p* < 0.01 compared to control group

### Neuroprotective effects of PUFA in OVX rats

**Fig. 2:**
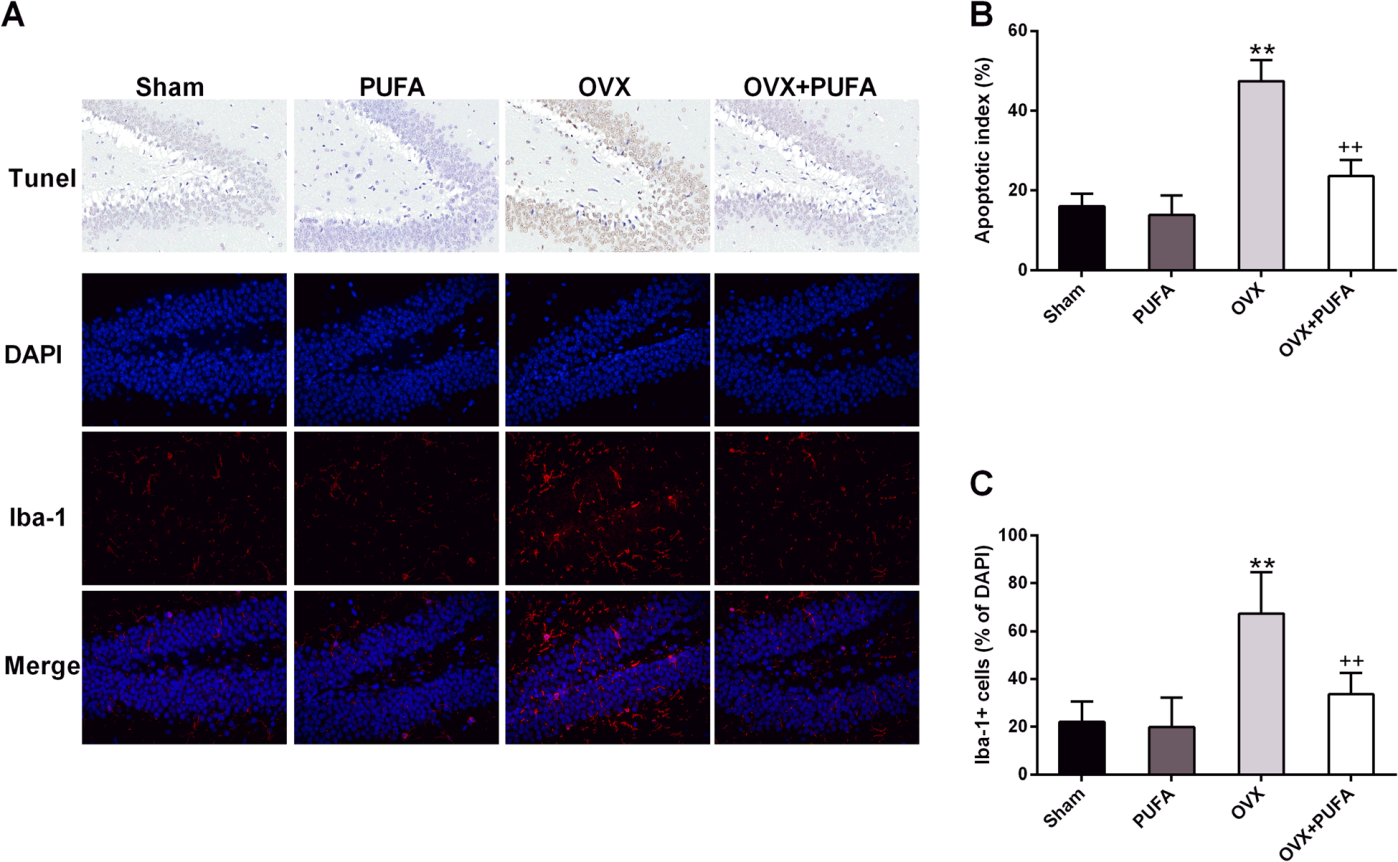
Neuroprotective effects of PUFA against OVX. Representative images of Tunel staining and immunofluorescence assays of Iba-1 in the hippocampal dentate gyrus (DG) region (**a**). Statistical graphs of apoptotic index (**b**) and Iba-1 positive cells (**c**). Data are means ± SD (*n* = 6–7). ^**^ *p* < 0.01 compared to Sham group. ^++^ *p* < 0.01 compared to OVX group

As shown in Fig. 2., chronic OVX induced the abundance of Tunel-positive cells compared with Sham group (main effect of OVX, F = 36.91, *p* < 0.01), indicating that the apoptotic cell rate in the hippocampus was significantly increased after OVX, whereas the rate of apoptotic cells in the OVX + PUFA group was markedly reduced compared with OVX group (PUFA×OVX interactions, F = 27.84, *p* < 0.01). Likewise, Iba-1 staining was used for the analysis of microglia activation [17] and two-way ANOVA revealed a significant interaction between PUFA and OVX (F = 15.63, *p* < 0.01). Post hoc comparisons demonstrated that PUFA treatment ameliorated OVX-induced microglia activation with a significant decrease of Iba-1 abundance (*p* < 0.01), suggesting potential anti-inflammatory effects.

### Effects of PUFA and OVX on cytokine expression and NF-κB signaling

**Fig. 3:**
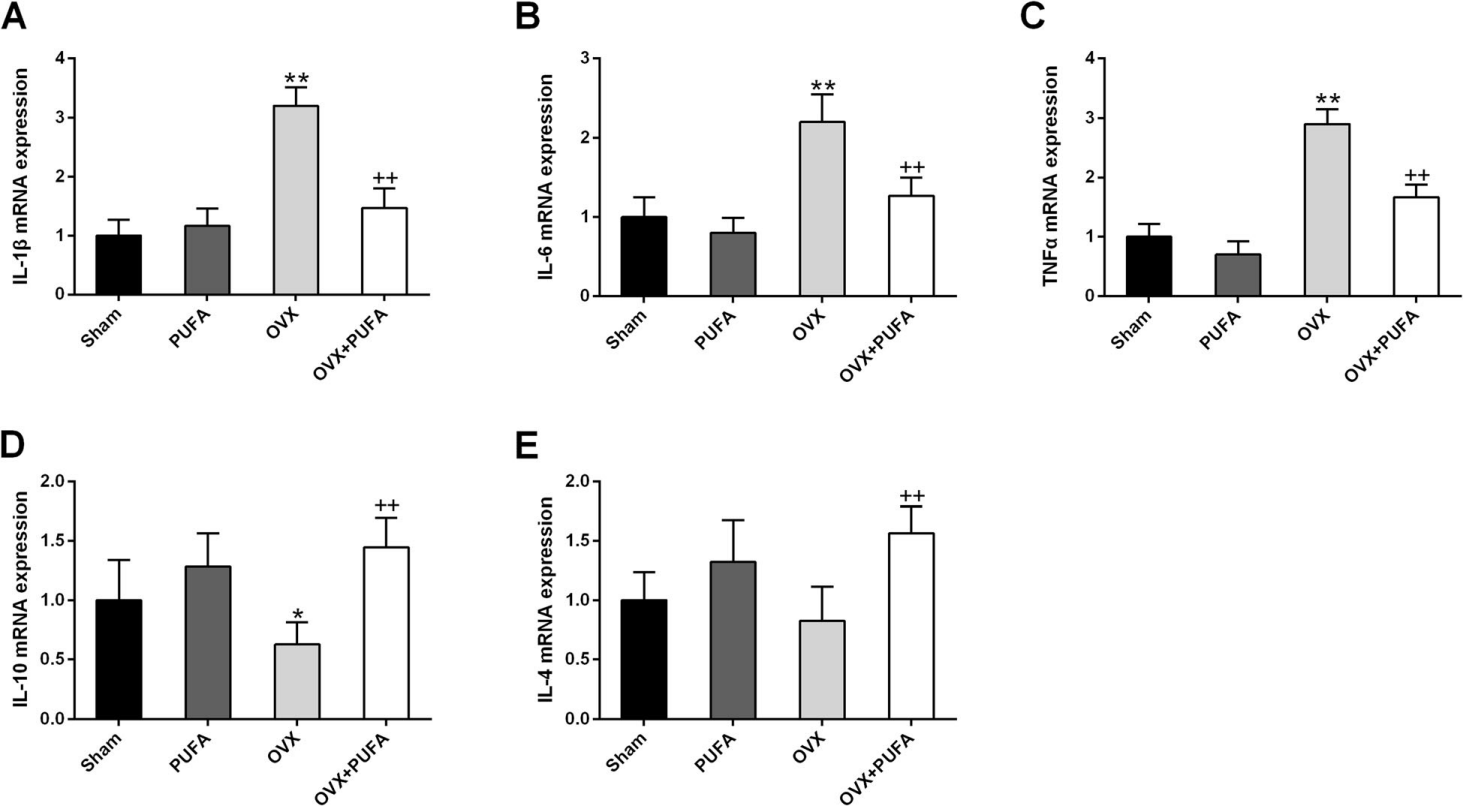
Anti-inflammatory effects of PUFA in OVX rats. Relative mRNA expression of proinflammatory cytokines, IL-1β (**a**), IL-6 (**b**) and TNFα (**c**), and anti-inflammatory cytokines, IL-10 (**d**) and IL-4 (**e**). Data are means ± SD (*n* = 6–7). ^*^ *p* < 0.05, ^**^ *p* < 0.01 compared to Sham group. ^++^ *p* < 0.01 compared to OVX group

**Fig. 4:**
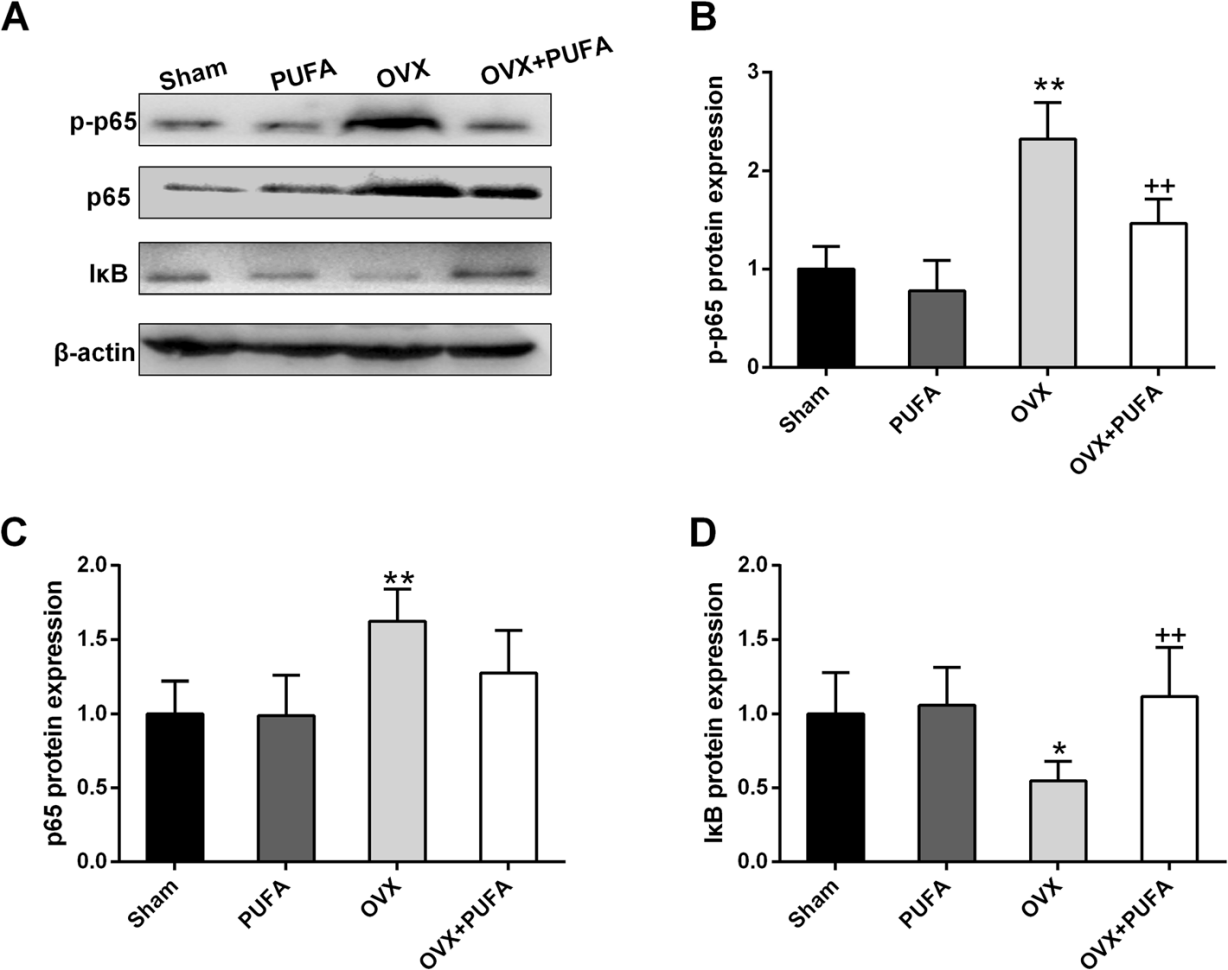
Effects of PUFA and OVX on NF-κB signaling. Representative blots (**a**) and statistical graphs of p-p65 (**b**), p65 (**c**) and IκB (**d**) expression. Data are means ± SD (*n* = 6–7). ^*^ *p* < 0.05, ^**^ *p* < 0.01 compared to Sham group. ^++^ *p* < 0.01 compared to OVX group

To further confirm the immune-regulatory effect of PUFA, the expression of inflammatory cytokines (Fig. 3) and NF-κB (Fig. 4) signaling were assessed. We observed a remarkable increase of pro-inflammatory cytokine expression (IL-1β, IL-6 and TNFα) (*p* < 0.01) but a marked decrease of anti-inflammatory IL-10 expression in the brain following the 10-week OVX (main effect of OVX, F = 6.24, *p* < 0.05). PUFA add-on treatment produced a reliable anti-inflammatory action by decreasing the expression of IL-1β, IL-6 and TNFα and increasing IL-10 and IL-4 expression in OVX animals. The transcription factor NF-κB serves as a dominated factor in coordinating the expression of a wide range of genes that control inflammatory responses [18]. We found that OVX decreased IκB protein expression (main effect of OVX, F = 5.93, *p* < 0.05) and increased the protein status of p-p65 (main effect of OVX, F = 32.51, *p* < 0.01) and p65 (main effect of OVX, F = 11.79, *p* < 0.01), whereas supplementation of PUFA suppressed the overactivated NF-κB signaling with a marked increase of IκB expression and a decrease of p65 phosphorylation level.

### Effects of PUFA and OVX on microglial polarization

**Fig. 5:**
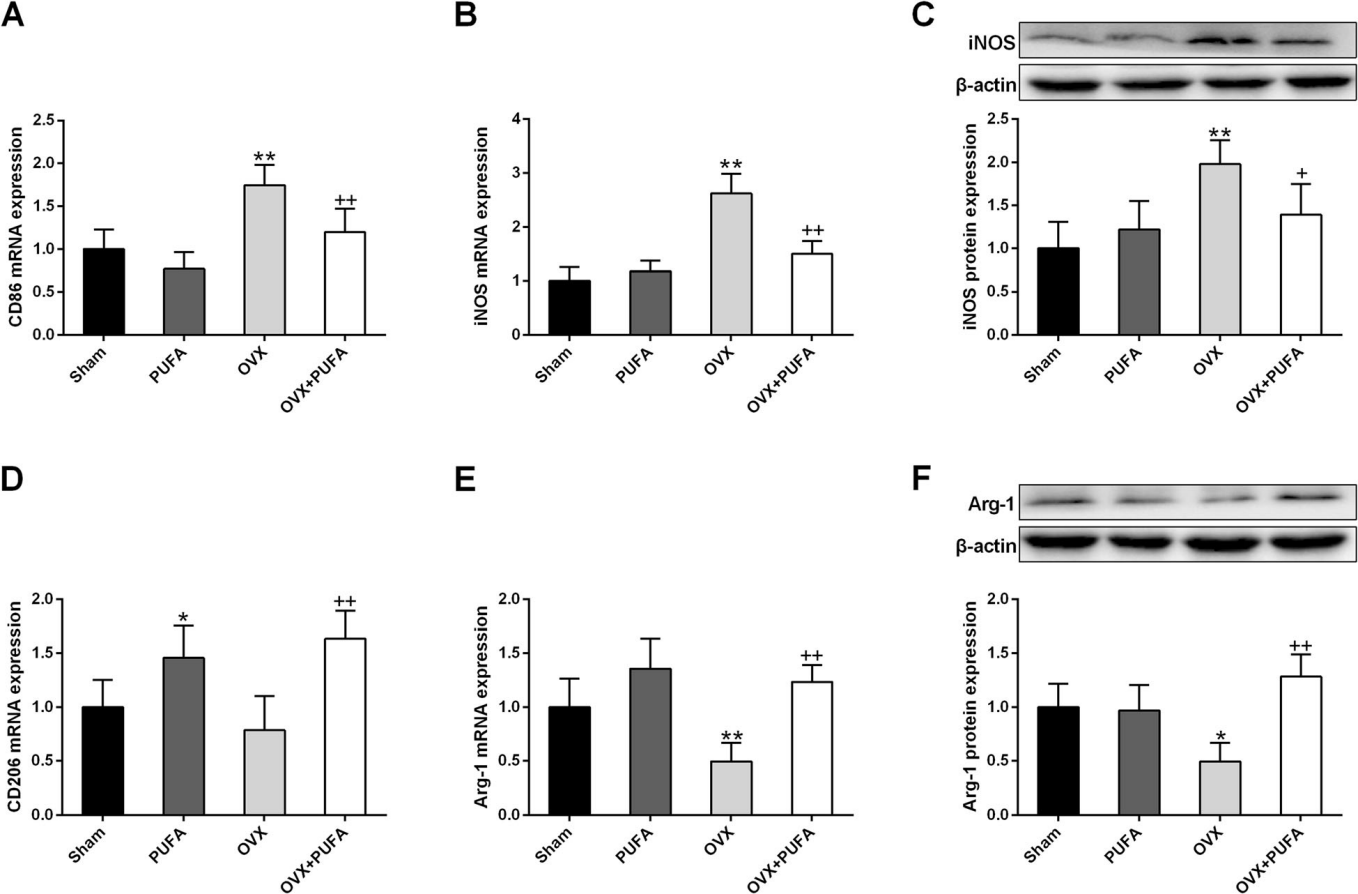
Effects of PUFA and OVX on microglial polarization. Relative mRNA expression of M1 microglial phenotype, CD86 (**a**) and iNOS (**b**), and protein expression of iNOS (**c**). Relative mRNA expression of M2 microglial phenotype, CD206 (**d**) and Arg-1 (**e**), and protein expression of Arg-1 (**f**). Data are means ± SD (*n* = 6–7). ^*^ *p* < 0.05, ^**^ *p* < 0.01 compared to Sham group. ^+^ *p* < 0.05, ^++^ *p* < 0.01 compared to OVX group

To further explore the microglial phenotype, the expression of biomarkers of proinflammatory M1 phenotype (CD86 and iNOS) and immunoregulatory M2 phenotype (CD206 and Arg-1) was analyzed [19]. In line with the proinflammatory environment caused by OVX, long-term OVX shifted the microglia from M2 to M1 phenotype with increased expression of M1 mediators but decreased expression of M2 biomarkers, which was also alleviated by co-treatment of PUFA during OVX state (Fig. 5).

## Discussion

Depression is more frequently observed in women, and menopause is a critical predisposing factor for the psychiatric disorders. Although estrogen replacement therapy is reported effective on the mental disorder progression, there is a great concern of the side-effects of chronic estrogen treatment, such as breast cancer and cardiovascular events [20, 21]. Therefore, it becomes urgent to explore the underlying mechanisms of menopause-related depression and seek an alternative treatment strategy for the mental condition.

Our data showed that chronic OVX induced anxiety-like and depression-like behaviors. Given previous research showed the time-dependent effects of OVX on behavioral changes that at least 6 weeks are needed for the animal to develop depression-like behaviors [22], the present study evaluated the behaviors and biochemical factors 10 weeks after OVX surgery to guarantee the animal model was successfully developed and PUFA was sufficiently supplemented. The experimental procedure succeeded as evidenced by the increased body weight and decreased uterine weight and estrogen levels. It should be noted that these basic characteristics were not changed by PUFA, indicating that the antidepressant effects is estrogen-independent. In support, it has been found that PUFA contains robust antidepressant effects in both clinical trials and chronic stress-induced depression-like rodents [23, 24]. Additionally, we previously found that PUFA administration mitigates neuroinflammation and depression-like behaviors in animals exposed to repeated LPS challenge [10]. In fact, the antidepressant effect of PUFA is, at least partially, attributed to its immune-modulating actions and the inflammatory progression is tightly linked to the depression-line behaviors caused by either chronic stress, LPS or OVX.

As expected, PUFA also ameliorated the neuronal apoptosis and neuroimmune overactivation caused by OVX as evidenced by the fact that the Tunel-positive cells and Iba-1 immunofluorescent abundance were remarkably decreased in OVX + PUFA group in comparison with OVX group. As previously reported [25], long-term following OVX induced the animals to a neuroimmune-activated condition with increased expression of proinflammatory cytokines but decreased expression anti-inflamamtory cytokine. The neuroinflammatory state is indicated to affect every pathological aspect of depression, including neurogenesis, neuronal apoptosis, neurotransmission and neuroplasticity, playing a major role in the onset and development of the mental disorder [26]. Microglia is the predominant resident immune cells in the brain responding to stressful stimuli. Generally, microglia can be categorized into two types, the classic pro-inflammatory M1 type and immunoregulatory M2 phenotype, which is responsible for the production of proinflammatory or anti-inflammatory cytokines, respectively. Although the switch of the M1/M2 microglial phenotypes is recently regarded as a novel therapeutic strategy for depression [27], there is no research evaluating microglial polarization in the OVX brain. The present research firstly showed that chronic OVX shifted the microglial polarization from M2 toward M1 phenotype as evidenced by the increased M1 biomarker (CD86 and iNOS) but decreased M2 biomarker (CD206 and Arg-1), which corresponded with the altered expression of inflammatory cytokines. Meanwhile, in accordance with the findings in the animal models of LPS or stress-induced depression [10], the anti-inflammatory and anti-depressant properties of PUFA were also verified in this OVX model. These beneficial effects are very likely to be associated with the phenotypic transition of microglia, as the present study showed that PUFA supplementation maintained the normal homeostatic balance between the M1 and the M2 phenotypes.

NF-κB serves as a dominated factor in coordinating the expression of a wide range of genes that control inflammatory responses and one of the proinflammatory mechanisms is that the activation of NF-κB signaling pathway is an essential trigger for the phenotypic transition from M2 to M1 phenotype [28]. Under normal physiological condition, NF-κB maintains in an inactive state in the cytoplasm, where it is bound to the inhibitor IκB. Once activated, IκB is phosphorylated and degraded, resulting in p65 phosphorylation and translocation, triggering inflammatory progression [29]. To provide the mechanistic insight, the key components of NF-κB pathway were assessed. The OVX procedures induced IκB degradation and enhanced the expression and phosphorylation of p65, indicating NF-κB signaling was activated, thereby shifting the microglial polarization from M2 toward M1 phenotype and promoting the transcription of proinflammatory cytokines. Intriguingly, PUFA co-treatment effectively re-balanced NF-κB signaling, preventing the pathway from overactivation induced by OVX. These results highlighted the involvement of NF-κB pathway underlying the anti-inflammatory and microglial regulatory mechanisms of PUFA in the condition of OVX.

## Conclusions

Collectively, the present study firstly showing the alterations of microglial polarization in OVX rats, shedding insight into the mechanisms of neuroinflammation and altered mood in menopause phase. Additionally, we found that PUFA also contains neuroimmune-regulatory and antidepressant effects in the context of OVX. These findings are interesting given accumulating evidence shows the protective actions of PUFA against cardiovascular hepatic dysfunction caused by OVX, providing novel evidence for the supplementary need of the multifunctional nutrient, PUFA, in menopause.

## Data Availability

The datasets used and analyzed during the current study are available fromthe corresponding author on reasonable request.

## Abbreviations

CA: Closed arms
CP: Central platform; FST forced swim test
ELISA: Enzyme-linked immunosorbent assay
EPM: Elevated plus maze
IL-1β: Interleukin -1β
IL-6: Interleukin − 6
LPS: Lipopolysaccharide
NSFT: Novelty-suppressed feeding test
OA: Open arms
OVX: Ovariectomized
PUFA: ω-3 polyunsaturated fatty acids
SPT: Sucrose preference test
TNFα: Tumor necrosis factor α
